# Molecular Preadaptation to Antimony Resistance in Leishmania donovani on the Indian Subcontinent

**DOI:** 10.1128/mSphere.00548-17

**Published:** 2018-04-18

**Authors:** F. Dumetz, B. Cuypers, H. Imamura, D. Zander, E. D’Haenens, I. Maes, M. A. Domagalska, J. Clos, J.-C. Dujardin, G. De Muylder

**Affiliations:** a Molecular Parasitology, Institute of Tropical Medicine, Antwerp, Belgium; b Advanced Database Reasearch and Modelling (ADReM), Department of Mathematics and Computer Science, University of Antwerp, Antwerp, Belgium; c Bernhard Nocht Institute for Tropical Medicine, Hamburg, Germany; d Department of Biomedical Sciences, University of Antwerp, Antwerp, Belgium; University of Texas Southwestern

**Keywords:** *Leishmania*, antimonials, drug resistance mechanisms, genomics, metabolomics

## Abstract

Antimonials (Sb) were used for decades for chemotherapy of visceral leishmaniasis (VL). Now abandoned in the Indian subcontinent (ISC) because of Leishmania donovani resistance, this drug offers a unique model for understanding drug resistance dynamics. In a previous phylogenomic study, we found two distinct populations of L. donovani: the core group (CG) in the Gangetic plains and ISC1 in the Nepalese highlands. Sb resistance was only encountered within the CG, and a series of potential markers were identified. Here, we analyzed the development of resistance to trivalent antimonials (Sb^III^) upon experimental selection in ISC1 and CG strains. We observed that (i) baseline Sb^III^ susceptibility of parasites was higher in ISC1 than in the CG, (ii) time to Sb^III^ resistance was higher for ISC1 parasites than for CG strains, and (iii) untargeted genomic and metabolomic analyses revealed molecular changes along the selection process: these were more numerous in ISC1 than in the CG. Altogether these observations led to the hypothesis that CG parasites are preadapted to Sb^III^ resistance. This hypothesis was experimentally confirmed by showing that only wild-type CG strains could survive a direct exposure to the maximal concentration of Sb^III^. The main driver of this preadaptation was shown to be *MRPA*, a gene involved in Sb^III^ sequestration and amplified in an intrachromosomal amplicon in all CG strains characterized so far. This amplicon emerged around 1850 in the CG, well before the implementation of antimonials for VL chemotherapy, and we discuss here several hypotheses of selective pressure that could have accompanied its emergence.

**IMPORTANCE** The “antibiotic resistance crisis” is a major challenge for scientists and medical professionals. This steady rise in drug-resistant pathogens also extends to parasitic diseases, with antimony being the first anti-*Leishmania* drug that fell in the Indian subcontinent (ISC). Leishmaniasis is a major but neglected infectious disease with limited therapeutic options. Therefore, understanding how parasites became resistant to antimonials is of commanding importance. In this study, we experimentally characterized the dynamics of this resistance acquisition and show for the first time that some *Leishmania* populations of the ISC were preadapted to antimony resistance, likely driven by environmental factors or by drugs used in the 19th century.

## Abstract (precis)

The “antibiotic resistance crisis” is a major challenge for scientists and medical professionals. This steady rise in drug-resistant pathogens also extends to parasitic diseases, with antimony being the first anti-*Leishmania* drug that fell in the Indian subcontinent (ISC). Leishmaniasis is a major but neglected infectious disease with limited therapeutic options. Therefore, understanding how parasites became resistant to antimonials is of commanding importance. In this study, we experimentally characterized the dynamics of this resistance acquisition and show for the first time that some *Leishmania* populations of the ISC were preadapted to antimony resistance, likely driven by environmental factors or by drugs used in the 19th century.

## INTRODUCTION

Leishmaniasis is one of the 17 neglected tropical diseases (NTDs) recognized by the WHO and is responsible every year for the death of about 30,000 persons in 98 countries where the disease is endemic, where 350 million people are at risk of contracting the disease (1). On the Indian subcontinent (ISC), leishmaniasis is present in its more lethal form, visceral leishmaniasis (VL). Its etiological agent, Leishmania donovani, is a dimorphic parasite transmitted by a phlebotomine vector (1, 2). Since 1923, pentavalent antimonials (Sb^V^) have been used to treat VL in the ISC and in other regions of the world. Sb^V^ is an immunomodulator active on the macrophage (3), and its reduced form, Sb^III^, is directly toxic for the parasite (4, 5). However, antimonials have lost their efficacy in the ISC toward the end of the 20th century, among others, because of drug resistance (DR) (6). In the ISC, antimonials were then replaced by miltefosine (MIL), but the efficacy of this drug is also declining (7): the first cases of MIL resistance were recently reported (8). A few drugs remain available (amphotericin B and paromomycin), and in the expectation of new compounds, it is essential to protect the current arsenal, among others, by using new formulations or combination therapy. Moreover, it is important to understand in depth the mechanisms and dynamics of DR.

A recent phylogenomic study of 204 L. donovani isolates shed new light on the emergence and spreading of antimonial-resistant (Sb-R) parasites in the ISC (9). First, the study described a natural history frame for the VL epidemics: the main population of L. donovani emerged about 150 years ago in northeast India, and after a succession of bottlenecks and expansions, it generated a series of subpopulations, currently defined as ISC2 to ISC10 and gathered within a main core group (CG). In addition, another genetic group (ISC1), which diverged much earlier from the CG, was discovered in the highlands of Nepal. Second, the study highlighted a series of features relevant in the context of Sb resistance. (i) All 191 isolates (Sb-R or Sb sensitive [Sb-S]) from the CG harbored two intrachromosomal amplifications (ICAs) of sets of adjacent genes, called the M and H loci; these genes were not amplified in isolates from the ISC1 subpopulation. The H locus was reported elsewhere to be a major driver of Sb^III^ resistance because of the presence of one gene, *MRPA*, encoding a member of the multidrug resistance protein family that sequesters Sb-thiol complexes into intracellular vesicles (10–12). (ii) Within the CG, a particular genetic group, ISC5, concentrates most of the strains isolated from antimonial treatment failure patients. All 52 strains of that group harbor a 2-nucleottide (nt) insertion in the AQP1 gene, creating a nonfunctional variant of this transporter known to be involved in the uptake of Sb^III^ (13, 14). (iii) Other genomic adaptations to Sb^III^, involved in detoxification mechanisms and increased levels of thiols reported in experimental resistance (15), had not been encountered (yet) in the natural population under study.

Considering the anthroponotic nature of VL in the ISC and the high rate of transmission documented in the Gangetic plains, we may assume that the CG represents the parasite population that has been under strong antimonial pressure over decades. The situation might be different in the highlands, where ISC1 was documented. In this region, VL cases were sporadic and L. donovani transmission has only been reported recently (16); hence we may hypothesize that antimonial pressure was historically lower in these regions than in the lowlands. In this context, the present study aimed at answering two main questions. First, do the dynamics of drug resistance emergence differ depending on the genetic background, known to be different between ISC1 and CG strains? Second, do we observe similar molecular adaptations when selecting antimonial resistance in the laboratory compared to natural selection in the field, and is the succession of events comparable?

These questions were answered, as a first step, in the context of Sb^III^ exposure. We first measured the intrinsic Sb^III^ susceptibility of promastigotes from 10 L. donovani strains representing the genetic diversity in the ISC. We then identified three reference strains among these (one from ISC1 and two from the CG) and experimentally selected Sb^III^ resistance at the promastigote stage. We analyzed two parameters: (i) the time to resistance (i.e., the number of weeks required upon stepwise selection to achieve resistance) and (ii) the type of resistance (i.e., the nature and dynamics of molecular adaptations developed by resistant parasites). This was done by untargeted genomic and metabolomic analyses and targeted transcript-level analyses. Validation of the main drivers of resistance was done by overexpression of candidate genes.

## RESULTS

### Intrinsic promastigote Sb^III^ susceptibility of L. donovani strains from ISC1 is higher than that of the core group.

**FIG 1:**
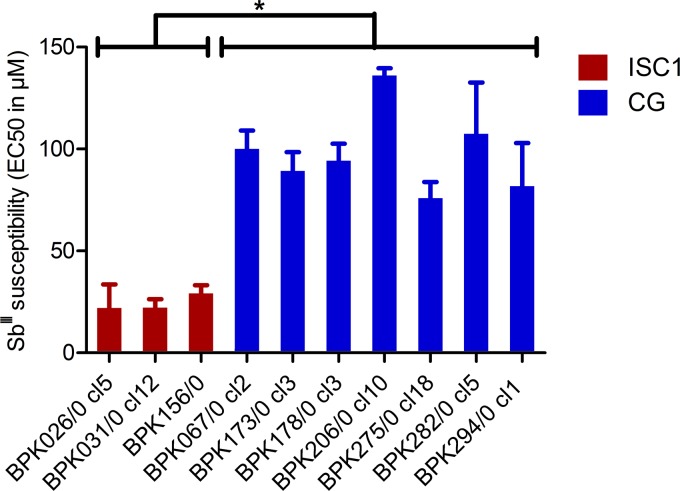
Sb^III^ susceptibility of promastigotes from ISC1 and core group (CG) strains. The three ISC1 strains (red) and the seven strains of CG (blue) are representative of the overall genetic diversity previously described in reference 9. Sb^III^ susceptibility for each strain was determined by three independent experiments using potassium antimony tartrate as an Sb^III^ source. *, *P* < 0.05 by ANOVA.

We first assessed the intrinsic Sb^III^ susceptibility of promastigotes representative of the genomic diversity encountered in Nepal (9). For this, we chose three cloned strains of the ISC1 group and seven strains of the CG (from the ISC3, -4, -5, -6, and -9 groups). The *in vitro* susceptibility assay revealed a similar 50% effective concentration (EC_50_) to Sb^III^ among CG promastigotes, with a mean value of 97.8 ± 19.9 µM; this contrasted with the different sodium stiboglucanate (SSG) susceptibility profiles observed for intracellular amastigotes of these strains shown in previous studies (9). In contrast, promastigotes from ISC1 were more susceptible to Sb^III^ than promastigotes from the CG, with a mean value of 24.4 ± 4 µM (Fig. 1; see Fig. S1 in the supplemental material). The difference observed between CG and ISC1 strains was statistically significant (*P*  = 3.2 × 10^−8^ by analysis of variance [ANOVA]).

10.1128/mSphere.00548-17.1 FIG S1  Percentage of parasite growth of BPK026 (red), BPK275 (light blue), and BPK282 (dark blue) when exposed to increasing doses of Sb^III^ for 24 h. Data are means from three replicates. Download FIG S1, TIF file, 0.5 MB. Copyright © 2018 Dumetz et al. 2018 Dumetz et al. This content is distributed under the terms of the Creative Commons Attribution 4.0 International license.

### Stepwise selection of Sb^III^ resistance in promastigotes.

**FIG 2:**
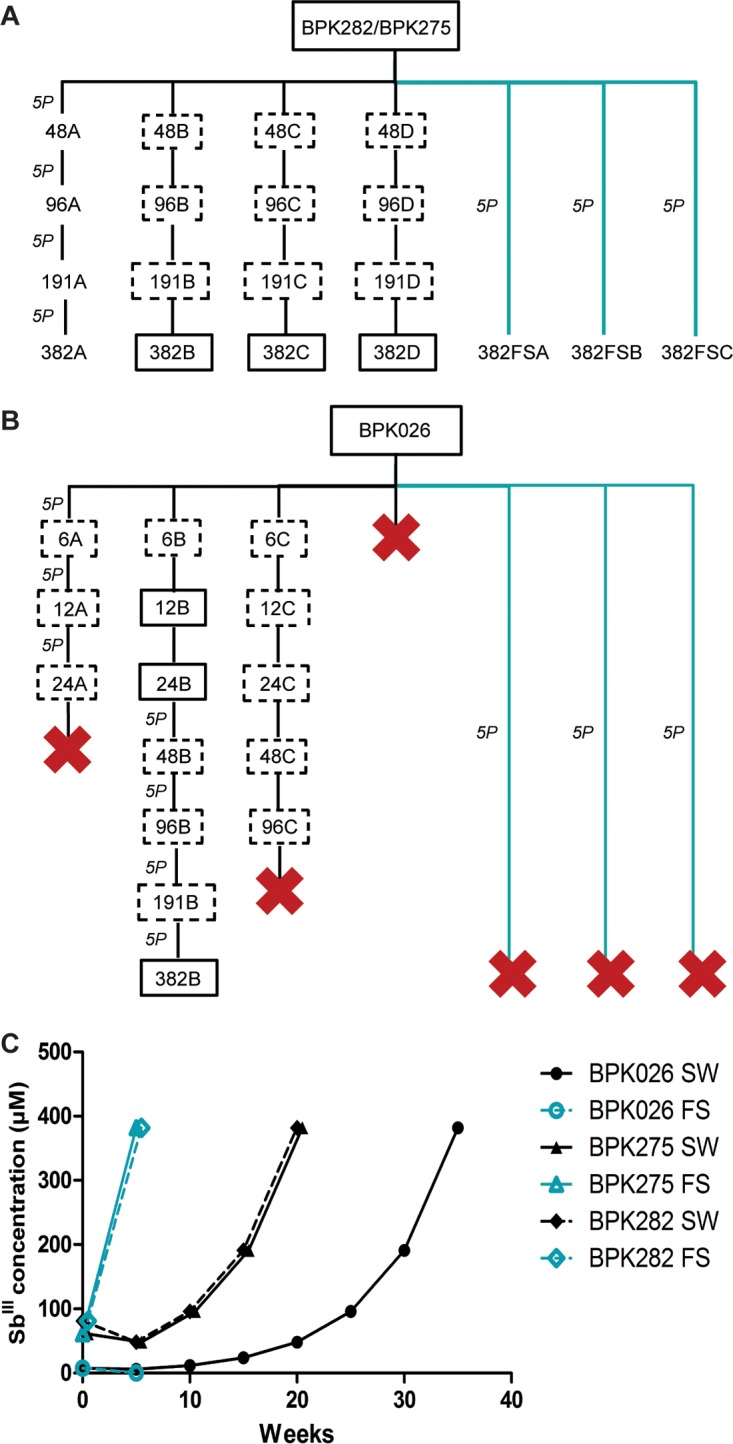
Dynamics of Sb^III^ resistance selection. (A and B) Flowchart of Sb^III^ resistance selection for CG strains BPK282 and BPK275 (A) and ISC1 strain BPK026 (B). Boxes indicate the increasing Sb^III^ concentrations (6 to 382 µM), and black lines are for the stepwise selection (SW) and blue lines for the flash selection (FS) of the different replicates (populations A to D). “5P” represents 5 *in vitro* passages. Red crosses show replicates that did not survive the selection pressure at a given drug concentration. Solid-line boxes represent samples analyzed at the genomic and metabolomics levels, and dashed-line boxes represent samples analyzed at the genomic level only. If no box is present, the samples were not analyzed. (C) Time to resistance in weeks for the 3 Sb^III^ stepwise-selected (SW [black lines]) and flash-selected (FS [blue lines]) strains.

We aimed to compare the dynamics of Sb^III^ resistance selection in three L. donovani strains differing in (i) intrinsic promastigote Sb^III^ susceptibility, (ii) intracellular amastigote Sb^V^ susceptibility (sensitive BPK026/282 versus resistant BPK275), and (iii) genomic background (see Table S1 in the supplemental material). The selection process was performed in quadruplicates (each replicate called line A, B, C, or D), starting with 47.75 µM Sb^III^ for both CG strains, a concentration close to their EC_50_. For BPK026, the selection process was initiated with 6 µM Sb^III^, as its EC_50_ was much lower. The two CG strains showed similar selection dynamics, with the four replicates of each strain successfully reaching the highest Sb^III^ concentration (382 µM) within four rounds of selection (Fig. 2A). These lines will be further called BPK282 Sb^III^-R and BPK275 Sb^III^-R. The selection dynamics for BPK026 were rather different: one replicate (line D) did not survive the first selection round, line A was cleared during the third round of selection (48 µM), and line C did not survive the sixth round (191 µM). Thus, one replicate only (line B) was successfully selected to survive the highest Sb^III^ concentration (further called BPK026 Sb^III^-R) (Fig. 2B). Time to resistance was defined as the time needed for each line to display a wild-type (WT) growth curve in the presence of 382 µM Sb^III^, following the stepwise selection process. Accordingly, time to resistance was estimated to 20 weeks for the two CG strains, whereas 35 weeks were required for BPK026 line B to reach the same level (Fig. 2C). In order to evaluate the stability of the resistance phenotype, all the Sb^III^-resistant lines as well as their parental strains were maintained for 20 weeks without Sb^III^. The withdrawal of the drug pressure did not impact their growth rate nor their Sb^III^ susceptibility (EC_50_, >382 µM) (data not shown).

10.1128/mSphere.00548-17.4 TABLE S1  L. donovani strains used. SSG activity indexes were calculated as previously described (S. Rijal et al., Microbes Infect 9:529–535, 2007, https://doi.org/10.1016/j.micinf.2007.01.009). An activity index of 1 corresponds to an EC_50_ ranging from 28.7 to 74 µM Sb^V^, while an activity index of 6 corresponds to an EC_50_ higher than 246.4 µM Sb^V^. Isolates with an activity index of 1 or 2 are considered sensitive, while those showing an activity index of 3 or more are considered resistant. Download TABLE S1, PDF file, 0.1 MB. Copyright © 2018 Dumetz et al. 2018 Dumetz et al. This content is distributed under the terms of the Creative Commons Attribution 4.0 International license.

To fully characterize the antimony resistance profile of the selected lines, we assessed the Sb^V^ susceptibility of intracellular amastigotes by applying SSG pressure on infected macrophages. Analysis of parental lines confirmed previous results: (i) intracellular amastigotes of both BPK026 and BPK282 clearly showed a dose-response curve typical of Sb^V^-sensitive strains, with EC_50_s of 42.46 and 27.51 µM, respectively (see Fig. S2 in the supplemental material); (ii) in contrast, for BPK275, similar infection indices were observed from 0 to 246.4 µM, characteristic of Sb^V^-resistant amastigotes. After Sb^III^ resistance selection of promastigotes, BPK275 Sb^III^-R amastigotes remained resistant to Sb^V^; BPK026 Sb^III^-R and BPK282 Sb^III^-R both showed a more than 6-fold-increased EC_50_ to Sb^V^ (276.7 and 170 µM, respectively) (Fig. S2).

10.1128/mSphere.00548-17.2 FIG S2  Dose-response curve of Sb^V^ against WT and Sb^III^-R intracellular amastigotes of BPK026, BPK275, and BPK282. Data are means from four replicates. Download FIG S2, TIF file, 0.7 MB. Copyright © 2018 Dumetz et al. 2018 Dumetz et al. This content is distributed under the terms of the Creative Commons Attribution 4.0 International license.

### Untargeted genomic analysis during stepwise Sb^III^ resistance selection.

**FIG 3:**
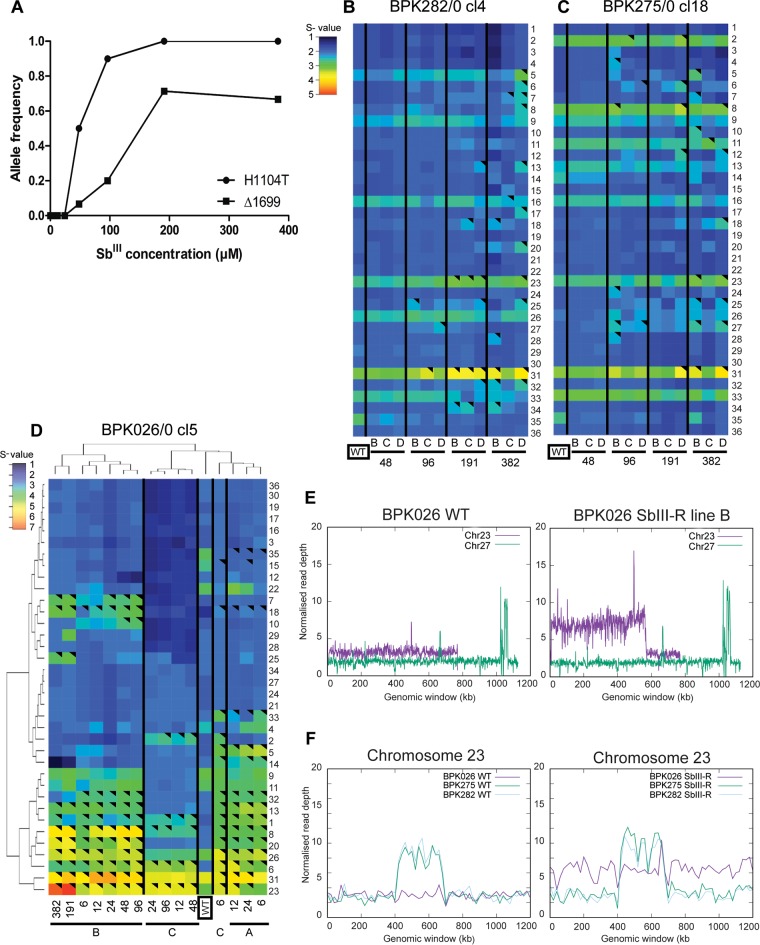
Genomic analysis of the selected Sb^III^-resistant strains. (A) Evolution of allele frequency over the Sb^III^ resistance selection process of BPK026 for a fusaric acid resistance protein-like protein (LdBPK_120011000-H1104T) and the intraflagellar transport protein (LdBPK_210017400-Δ1699). (B and C) Heat map representing the karyotype dynamics across the Sb^III^ resistance selection of BPK282 and BPK275. (D) High hierarchical representation of BPK026 karyotype evolution. Heat maps show median normalized read depths of chromosomes found within each cell population for each of the 36 chromosomes (*y* axis) and each sample (*x* axis), replicate populations called A, B, C, and D are shown for each selecting Sb^III^ concentration (from 6 to 382 µM), and the samples “WT” framed in a black box represent the parental Sb^III^-sensitive population for each strain. The color key for panels B and C shows the normalized chromosome read depth (*S*). *S* ranges are as follows: monosomy, *S* < 1.5 (dark blue); disomy, 1.5 ≤ *S* < 2.5 (light blue); trisomy, 2.5 ≤ *S* < 3.5 (green); tetrasomy, 3.5 ≤ *S* < 4.5 (orange); pentasomy, 4.5 ≤ *S* < 5.5 (red). In panel D, the ranges are as follows: monosomy, *S* < 1.5 (dark blue); disomy, 1.5 ≤ *S* < 2.5 (blue); trisomy, 2.5 ≤ *S* < 3.5 (light blue); tetrasomy, 3.5 ≤ *S* < 4.5 (green); pentasomy, 4.5 ≤ *S* < 5.5 (yellow); hexasomic, 5.5 ≤ *S* < 6.5 (orange); heptasomic, 6.5 ≤ *S* < 7.5 (red). A black triangle in an upper right corner indicates a significant change in *S* value (>0.5, with a shift from one *S* range to another and a *P* value of ≤10^−5^) in comparison to the *S* value of the starting population (sample “0” framed in a black box). (E) The left panel shows the normalized read depth of chromosome 23 (trisomic) compared, as a control, to the normalized read depth of chromosome 27 (disomic) in the parental strain BPK026 (BPK026 WT), and the right panel shows the normalized read depth for these two chromosomes in BPK026 Sb^III^-R. (F) Normalized read depth of the fragment of chromosome 23 harboring the H locus. The left panel displays the parental strains (BPK026 WT, BPK275 WT, and BPK282 WT), while the right panel shows Sb^III^-R line B for the three isolates.

For all parasite lines, samples were collected at each selection round for whole-genome sequence analysis of nuclear DNA (see Fig. 2A and B for details). We first looked at the DNA sequence itself to detect single nucleotide polymorphisms (SNPs) and small nucleotide insertions or deletions (indels). The average coverage of all the analyzed samples was 13.5× ±3.9, ranging from 7.4 to 24.9×, and >98% of the genome was covered. All along the selection process, the two CG strains did not show any *de novo* SNP or indel in the whole nuclear genome; among 303 heterozygous sites in the CG strains, no significant changes in allele frequency were detected. In BPK026 Sb^III^-R line B, we did not observe any new indel during the selection process, but in two different genes, a heterozygous SNP appeared at round 4, under an Sb^III^ pressure of 48 µM. These two *de novo* SNPs, respectively, introduced a stop codon at position 1699 of LdBPK_210017400 (formerly LdBPK_211220.1), coding for an intraflagellar transport protein, and a nonsynonymous mutation, H1104T, in LdBPK_120011000 (formerly LdBPK_120530), coding for a fusaric acid resistance protein-like protein. In subsequent selection rounds, the frequency of the mutated alleles progressively increased together with the Sb^III^ concentration (Fig. 3A), from 0.067 to 0.667 (LdBPK_210017400) and from 0.5 to 1 (LdBPK_120011000). Among the 11,189 heterozygous sites of BPK026, no change in allele frequency was observed during the selection process.

In a second stage, we looked at genome structure changes, more specifically somy and local copy number variations (CNVs), both known to occur when *Leishmania* is under selective pressure. The karyotype variability contrasted sharply with the conservation of the nucleotidic sequence itself reported above. However, this variability was much higher in BPK026 than in the two CG strains, with 153 significant somy changes among all lines of BPK026 versus 31 and 28 significant changes in BPK282 and BPK275, respectively (Fig. 3B, C, and D). Interestingly, at each round of selection, karyotypes of distinct replicate lines were rather similar for the CG strains, while different “scenarios” of aneuploidy were followed by the three replicate lines of BPK026. Five chromosomes (8, 18, 23, 25, and 31) showed an increase in the statistical significance of chromosome copy number (*S* value) in one or more lines of each strain, the most extensive increases being observed for chromosomes 23 and 31 (Fig. 3; see Data Set S1 in the supplemental material).

10.1128/mSphere.00548-17.6 DATA SET S1  Quantitative changes in *S* value observed in Fig. 3B, C, and D. Download DATA SET S1, XLSX file, 0.1 MB. Copyright © 2018 Dumetz et al. 2018 Dumetz et al. This content is distributed under the terms of the Creative Commons Attribution 4.0 International license.

Interestingly, the *S* value increase of chromosome 23 in BPK026 Sb^III^-R was due to a strong increase in the read depth of a large chromosomal segment (from positions 10048 to 567768 [Fig. 3E]): at 382 µM, this fragment showed a copy number of 7.1 ± 1.3, while the small segment (from positions 569158 to 768383) showed a copy number similar to that of the WT (3.0 ± 0.4). These additional copies of the large segment of chromosome 23 resulted in the specific amplification of 140 open reading frames (ORFs), of which 52.85% are putatively annotated and 47.15% correspond to hypothetical proteins. Interestingly, among these genes, 6 (3.8%) were coding for proteins involved in redox pathways, while none of these were found in the small fragment. In addition, several genes of the large fragment have been previously reported to be involved in (i) drug resistance or (ii) virulence. (i) The large fragment genes associated with drug resistance were LdBPK_230007200 and _7300 (previously LdBPK_230230.1), coding for pentamidine resistance protein, LdBPK_230007700 and _7800 (previously LdBPK_230280 and _290, respectively), associated with the terbinafine resistance locus and MRPA within the H locus, and LdBPK_230009800 (previously LdBPK_230500), coding for trypanothione synthetase. (ii) The large fragment genes associated with virulence were LdBPK_230018500 and _18600, encoding hydrophilic acylated surface proteins a and b (previously not assembled), and LdBPK_230020400 (previously LdBPK_231430), coding for membrane-bound acid phosphatase 2. All other protein categories analyzed by Gene Ontology were represented in comparable proportions in both fragments (Data Set S2A and B).

10.1128/mSphere.00548-17.7 DATA SET S2  Gene Ontology analysis for chromosome 23. Download DATA SET S2, XLSX file, 0.1 MB. Copyright © 2018 Dumetz et al. 2018 Dumetz et al. This content is distributed under the terms of the Creative Commons Attribution 4.0 International license.

Local CNVs like the H and M loci, which are constitutive of the CG (9), did not vary in copy number in the CG lines: they also did not appear as intra- or extrachromosomal amplifications in BPK026 Sb^III^-R (Fig. 3F). Altogether, the untargeted genomic analysis revealed that along the selection process, BPK026 developed more molecular adaptations than the two CG strains, including amplification of genes known to be involved in drug resistance and virulence.

### Targeted transcript-level analysis of Sb^III^-resistant selected lines and validation of main drivers.

**FIG 4:**
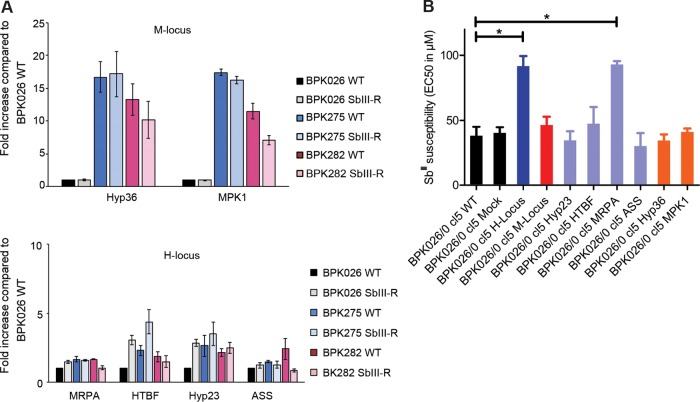
Importance of H and M loci for Sb^III^ resistance. (A) Expression levels of genes of the M locus (above) and H locus (below) in parental lines (BPK026 WT, BPK282 WT, and BPK275 WT) and one respective Sb^III^-resistant population (BPK026 Sb^III^-R pop B, BPK282 Sb^III^-R pop B, and BPK275 Sb^III^-R pop B) were measured by qRT-PCR. Data were first normalized to the stable *SAT* gene and LdBPK_240021200 genes and then rescaled to the levels of BPK026 WT. Data are means ± standard deviation (SD) from two independent experiments. (B) Sb^III^ susceptibility of the BPK026 WT and BPK026 WT transfected with the H or M locus or with the individual genes of these loci. Data are means from three independent experiments. *, *P* < 0.05 by ANOVA.

Given the general amplification of the H and M loci in the CG strains (9, 17), as well as the observed changes in chromosome 23 during the Sb^III^ selection process, we performed quantitative reverse transcription (RT)-PCR assays to compare the expression levels of genes present in the H and M loci in the parental and Sb^III^-resistant lines generated here. With respect to the M locus, we observed a higher expression of the two tested genes (Hyp36 and MPK1) in parental (WT) CG lines versus BPK026, which reflected the intrachromosomal amplification of the M locus; however, Sb^III^ resistance in both BPK026 and the CG lines was not accompanied by a further increase of the expression of these genes (Fig. 4A). The H locus was the most interesting target; as for the M locus, levels of MRPA, HTBF, Hyp23, and ASS transcripts were slightly higher in WT CG lines than in WT BPK026 (1.5- to 3.0-fold increase), which also reflected the intrachromosomal amplification of the H locus in CG lines. After selection of Sb^III^ resistance, there were no significant changes in the expression of these genes within the CG lines, but in BPK026, the levels of the four genes increased 1.5- to 3.5-fold, reaching the expression levels of the CG lines (Fig. 4A). This fit with the increased copy number observed for the large fragment of chromosome 23 (harboring among others the H locus) mentioned above.

In order to confirm the importance of the H locus and its amplification for Sb^III^ resistance (through intrachromosomal amplification or partial increase of chromosome 23’s genomic material), the full H locus was overexpressed in BPK026 promastigotes using a cosmid carrying that region; similar overexpression of the M locus was done as control. This experiment revealed that overexpression of the H locus was associated with an increase of Sb^III^ tolerance of about 3-fold (*P* = 6.6 × 10^−3^ by ANOVA), in contrast to overexpression of the M locus, which did not impact drug susceptibility. We further dissected the importance of the different genes constituting both H and M loci, by overexpressing them separately in BPK026. For one gene only, *MRPA*, overexpression was associated with increased Sb^III^ tolerance (3-fold, *P* = 1.71 × 10^−3^ by ANOVA) comparable to that of the CG isolates (Fig. 4B; see Fig. S3 in the supplemental material).

10.1128/mSphere.00548-17.3 FIG S3  Difference in Sb^III^ susceptibility of BPK026 overexpressing MRPA or the H locus (orange) and CG strains (blue). The black star indicates significant difference from the BPK026-H-Locus strain, and the red star indicates significant difference from the BPK026/0-MRPA strain (*P* < 0.05 by ANOVA). Download FIG S3, TIF file, 2.9 MB. Copyright © 2018 Dumetz et al. 2018 Dumetz et al. This content is distributed under the terms of the Creative Commons Attribution 4.0 International license.

### Untargeted metabolomics analysis of Sb^III^-resistant selected lines.

**FIG 5:**
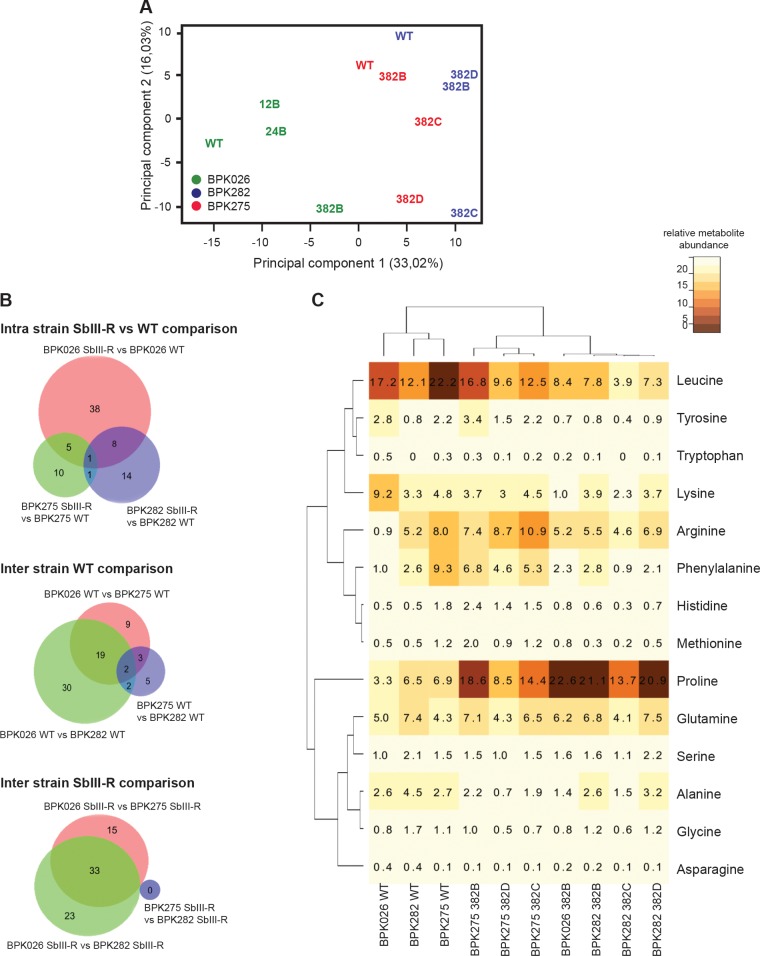
Metabolomics analysis of the selected Sb^III^-resistant strains. (A) Principal-component analysis based on 300 metabolites and including the initial parental strains (WT) BPK026 (green), BPK275 (red), and BPK282 (blue), the final populations resistant to 382 µM Sb^III^ (BPK026 382B, BPK275 382B, -C, and -D, and BPK282 382B, -C, and -D) as well as BPK026 intermediates (12B and 24B, respectively, resistant to 12 and 24 µM Sb^III^). (B) Venn diagrams showing metabolites significantly different between strains. (C) High hierarchical representation of amino acid abundance in the different strains, including the parental strains (BPK026 WT, BPK282 WT, and BPK275 WT) and the selected populations resistant to 382 µM Sb^III^ (BPK275 382B, -C, and -D, BPK026 382B, and BPK282 382B, -C, and -D).

An untargeted metabolomics analysis was performed on the three parental lines, their respective fully resistant lines (382 µM), and several intermediate resistant lines (12 and 24 µM) of BPK026 line B. In total, we detected 300 metabolites (see Data Set S3A in the supplemental material). Principal-component analysis (PCA) was carried out as a first exploratory analysis of this metabolomics data set (Fig. 5A). The two first principal components (PC1 and -2) explained 33.02 and 16.03% of the total variation and clearly separated two experimental sets: (i) the BPK026 parental and resistant lines and (ii) the BPK275 parental and resistant lines B, C, and D, together with BPK282 parental and resistant lines B, C, and D. For BPK026, the two intermediate-resistant populations clustered at an intermediate position between the parental strain and the fully resistant line. PCA results were confirmed by analysis of individual metabolites. The numbers of metabolites significantly differing between parental and the fully resistant lines ranged as follows: 52 (BPK026), 24 (BPK282), and 17 (BPK275), highlighting the larger amount of changes occurring in the BPK026 Sb^III^-R strain (Fig. 5B; Table 1). A comparison of all the resistant lines showed the metabolic similarity of the CG strains BPK282 and BPK275 (Fig. 5A and B; Data Set S3A and C). This general trend was confirmed when analyzing specific classes of metabolites; among them two classes represented significant common changes during all the comparisons we performed—glycerophospholipids (GPLs) and amino acids.

10.1128/mSphere.00548-17.8 DATA SET S3  Metabolomics data. Table A shows metabolite abundances and log_2_ fold changes. Table B shows differential metabolite abundance comparing parental strains. Table C shows differential metabolite abundance comparing Sb^III^-R lines. Download DATA SET S3, XLSX file, 0.5 MB. Copyright © 2018 Dumetz et al. 2018 Dumetz et al. This content is distributed under the terms of the Creative Commons Attribution 4.0 International license.

**TABLE 1:** Differential metabolite abundance between Sb^III^-R and parental lines of each of the three strains considered here

Metabolite class	No. and name(s) of metabolites from^a^:		
	BPK026 Sb^III^-R/BPK026 WT	BPK282 Sb^III^-R/BPK282 WT	BPK275 Sb^III^-R/BPK275 WT
Increased			
Amino acids, peptides, and amino acid conjugates	9: arginine, proline, isoleucine, glutamylamino-butanoate, *N*^2^-succinyl-ornithine, glutamylcysteine, prolyl-proline, phenylalanine, glutamylalanine	1: proline	2: acetyl-lysine, prolylhydroxyproline
GPLs	7: 1 saturated or low unsaturated, 6 highly unsaturated	3: 1 saturated or low unsaturated, 2 highly unsaturated	
Thiols	1: glutathione		
Nucleobases and nucleosides		1: xanthine	
Others	23: flumipropyn; magnoshinin; clobenpropit; PC(20:4(5*Z*,8*Z*,11*Z*,14*Z*)/18:4(6*Z*,9*Z*,12*Z*,15*Z*)); 2′-hydroxyfurano [2″,3″:4′,3′]chalcone; coenzyme M 7-mercaptoheptanoylthreonine-phosphate heterodisulfide; PC(22:5(4*Z*,7*Z*,10*Z*,13*Z*,16*Z*)/22:5(4*Z*,7*Z*,10*Z*,13*Z*,16*Z*)); 5-hydroxy-6,7,3′,4′,5′-pentamethoxyflavanone 5-*O*-rhamnoside; *N*^4^-phosphoagmatine; (*S*)-AMPA; 5-l-glutamylglycine; uplandicine; 1-pyrroline; piperidine; 4-aminopyridine; histamine; γ-Glu–Thr; carnitine; dihydro-4,4-dimethyl-2,3-furandione; hexitol; 3,4,5-trihydroxy-hexanoic acid, rhamnose, deoxygalactopyranose, fucose 1,5-anhydrosorbitol; methylglutaric acid, adipic acid, solerol, 2,2-dimethylsuccinic acid, aceto-hydroxybutanoic acid; xylitol, arabitol; 4-guanidinobutanal	2: 5-hydroxy-6,7,3′,4′,5′-pentamethoxyflavanone 5-*O*-rhamnoside; 1-pyrroline	2: 7-oxo-8-amino-nonanoic acid 8-amino-7-oxo-nonanoic acid; 2-ethyl-2-hydroxybutyric acid, 2-hydroxy-3-methylpentanoic acid, hydroxy(iso)caproic acid, X-hydroxy-hexanoic acid
Total	40	7	4

Decreased			
Amino acids, peptides, and amino acid conjugates	2: proline-betaine; trimethyl-lysine	4: glutamylalanine; succinyldiaminopimelate, *N*^2^-succinyl-l-arginine; X-amino-pentanoic acid, valine	3: isoleucine, glutamylcysteine, argininosuccinate
GPLs	5: 4 saturated or low unsaturated, 1 highly unsaturated		1: phosphorylethanolamine
Thiols		1: trypanothione-disulfide	
Nucleobases and nucleosides			1: IMP
Others	5: creatine; cyclohexane-1,3-dione; spisulosine, 1-deoxy-sphinganine; 2-keto-3-methyl-valerate, 2K-4CH_3_-pentoate, X-oxo-X-methyl-pentanoic acid, 2-ketoisocaproate, X-oxo-hexanoic acid; *S*-adenosylmethionine	12: allantoin; 2-ethyl-2-hydroxybutyric acid, 2-hydroxy-3-methylpentanoic acid, hydroxy(iso)caproic acid, X-hydroxy-hexanoic acid; *N*-acetylglutamine; γ-Asp–Asp–Pro; 5-l-glutamylglycine; clobenpropit; guvacine; (*S*)-AMPA; cholinephosphate; fagomine; dehydroalanine; SP(34:0)	8: flumipropyn; glucolepidiin; 5-hydroxy-6,7,3′,4′,5′-pentamethoxyflavanone 5-*O*-rhamnoside; γ-Lys–Val; PC(22:5(4*Z*,7*Z*,10*Z*,13*Z*,16*Z*)/22:5(4*Z*,7*Z*,10*Z*,13*Z*,16*Z*)); PC(22:5(4*Z*,7*Z*,10*Z*,13*Z*,16*Z*)/20:2(11*Z*,14*Z*)); 1-eicosanoyl-2-(4*Z*,7*Z*,10*Z*,13*Z*,16*Z*,19*Z*-docosahexaenoyl)-sn-glycero-3-phosphocholine; coenzyme M 7-mercaptoheptanoylthreonine-phosphate heterodisulfide
Total	12	17	13

a Only the biologically (2-fold changes) and statistically (*P* < 0.05) significant differences are indicated.

GPL composition evolved differently over the selection process of the different strains: we observed 13 changes between BPK026 WT and BPK026 Sb^III^-R, while only four changes were detected in BPK282 Sb^III^-R compared to the BPK282 WT, and lipid composition was unchanged between all BPK275 lines. When the GPL composition of the BPK026 WT was compared to the two WT strains of CG taken together, we observed smaller amounts of 10 GPLs in the former (three unsaturated/low unsaturated and seven highly unsaturated), and 3 were more abundant (low unsaturated). When doing the same comparison between resistant lines of the three strains, no difference was observed (Table 2). This indicated that upon Sb^III^ resistance selection of BPK026, the levels of 13 GPLs evolved toward levels encountered in CG lines.

**TABLE 2:** Changes in glycerophospholipid abundance

GPL type	Change in abundance for comparison shown^a^											
	BPK026 Sb^III^-R^b^ vs BPK026 WT		BPK282 Sb^III^-R^c^ vs BPK282 WT		BPK275 Sb^III^-R^c^ vs BPK275 WT		BPK026 WT vs BPK282 WT		BPK026 WT vs BPK275 WT		BPK026 Sb^III^-R vs CG Sb^III^-R^d^	
	Up	Down	Up	Down	Up	Down	Up	Down	Up	Down	Up	Down
Saturated and low unsaturated (≤3)	0	1	1	0	0	0	0	3	0	0	0	0
High unsaturated (>3)	8	4	3	0	0	0	0	4	3	3	0	0

a “Up” represents a log_2_ FC of >1, and “Down” represents a log_2_ FC of <1.

b BPK026 Sb^III^-R population B.

c The values shown for BPK282 and BPK275 Sb^III^-R are the average values from the three resistant replicates (populations B, C, and D).

d The value shown for CG Sb^III^-R is the average of the values for BPK282 and BPK275 Sb^III^-R replicates.

Amino acids were also significantly altered in the current experimental set. Again, more amino acids changed during Sb^III^ resistance selection of BPK026 (*n =* 4) than in BPK282 (*n =* 2) and BPK275 (*n =* 2). These 4 amino acids deserved particular attention due to their variation in abundance as well as their biological importance. Two amino acids varied in all resistant lines versus their WT parental strain: (i) proline abundance increased 6.76-fold in BPK026 Sb^III^-R, 2.8-fold on average in BPK282 Sb^III^-R, and 2-fold in average in BPK275 Sb^III^-R lines; (ii) leucine abundance decreased 2-fold in BPK026 Sb^III^-R, 1.9-fold in BPK282 Sb^III^-R lines, and 1.7-fold in BPK275 lines. Arginine was less abundant in the BPK026 WT compared to the CG strains (5.7-fold decrease compared to the BPK282 WT and 8.8-fold decrease compared to the BPK275 WT); interestingly, the levels of arginine did not change significantly during the Sb^III^ selection of CG lines, but it increased 5.7-fold in BPK026 Sb^III^-R versus the WT parental strain (Fig. 5C; Data Set S3A), thus reaching the levels encountered in CG lines. Finally, lysine was more abundant in the BPK026 WT than in the CG WT strains and dropped drastically (9.2-fold) in BPK026 Sb^III^-R, while levels did not change in CG resistant lines.

### Adaptation of L. donovani strains to a high concentration of Sb^III^.

**FIG 6:**
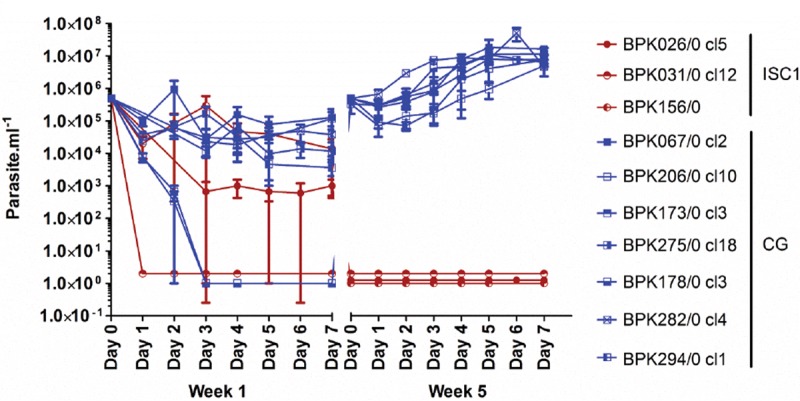
Flash selection with 382 µM Sb^III^ on ISC1 and CG strains. Shown are growth curves over 5 weeks of three ISC1 strains (red) and seven CG strains (blue) in the presence of 382 µM Sb^III^. Each point represents the average value of promastigote counts in three independent flasks.

The previous results suggested that promastigotes of CG strains are better prepared than BPK026 to respond to Sb^III^ exposure. To test this preadaptation hypothesis under extreme conditions, we assessed the possibility for the parasites to survive the highest concentration of Sb^III^ without the stepwise selection previously undertaken. The 10 strains described above and representing ISC1 (BPK026, BPK156, and BPK031) and CG (BPK067, BPK206, BPK275, BPK173, BPK282, BPK178, and BPK294) (Fig. 1; Table S1) were used for a “flash selection,” meaning direct exposure of a wild-type strain to 382 µM Sb^III^ during 5 weeks, passaging the parasites every 7 days and performing a growth curve for 5 weeks. Interestingly we observed from the first day a drastic reduction of parasite numbers for all strains and already a complete loss of BPK031 (ISC1). The two other ISC1 strains suffered of a drastic decrease, but living parasites could still be observed after 7 days in at least one replicate. After 5 weeks, no living parasites were detected in any of the three ISC1 strains. In sharp contrast, all seven CG strains recovered their original growth rate after four passages only (Fig. 2A and C and 6).

## DISCUSSION

The objective of our study was to analyze experimentally the dynamics of emergence of Sb^III^ resistance in ISC strains from different genetic backgrounds, to characterize molecular adaptations developed by resistant lines, and to compare them to the adaptations encountered in a clinical context. Our study was performed *in vitro* with promastigotes and the reduced form of antimony, Sb^III^. As such, it does not *a priori* take into account resistance mechanisms related to the immunomodulatory effect of Sb^V^ nor the reduction of Sb^V^ to Sb^III^, but mechanisms related to transport and detoxification of Sb^III^. This study therefore constitutes a first step in the analysis of *Leishmania* adaptations to antimonials but should be complemented in the future by similar work with amastigotes and Sb^V^.

We showed here that (i) Sb^III^ susceptibility of promastigotes was higher in ISC1 than in the CG (Fig. 1; Table S1); (ii) following a classical drug resistance selection scheme, time to Sb^III^ resistance (Fig. 2C) was higher for ISC1 parasites (35 weeks) than for CG strains (20 weeks); (iii) untargeted genomic and metabolomic analyses revealed that molecular changes associated with the acquisition of Sb^III^ resistance were more numerous in the ISC1 strain than the CG strain; and (iv) during selection, genomic changes were very similar in the replicates of each CG strain, while very different scenarios were observed among replicates of ISC1, only one of them leading to full resistance. Altogether, these observations led to the hypothesis that CG promastigotes are preadapted to Sb^III^ resistance. This hypothesis was experimentally tested by a “flash selection”—i.e., a direct exposure of WT parasites to the maximal concentration of Sb^III^ (Fig. 6; Table S1). The fact that none of the ISC1 strains survived the flash selection while all the CG strains could recover growth comparable to that of the WT after 5 weeks confirmed the preadaption hypothesis.

Interestingly, based on our phylogenomic study of heterochronous isolates, we estimated the emergence of the Indo-Nepalese populations of the CG around 1900: this was revealed by a Bayesian phylogenetic model in an explicit temporal and spatial framework (9) and supported by historical reports (18). This corresponds more or less to the first use of Sb^III^ in clinical practice (1919), before being replaced by Sb^V^ in 1923 because of toxicity (9). In the anthroponotic context of L. donovani transmission in the ISC, it is possible that these 4 years of application of Sb^III^ provided the selective pressure to the Indo-Nepalese ancestors of the CG. However, there are two alternative explanations. On one hand, the widespread contamination of drinking water by arsenic, shown to occur in the ISC (19), could have provided a strong selective pressure. Arsenic and antimonials share the same sequestration mechanism via MRPA overexpression (12). Furthermore, it was demonstrated that L. donovani could develop resistance to sodium stibogluconate (Pentostam) after exposure to arsenic (20). On the other hand, considering (i) the importance of the H locus amplification in the preadaptation here reported, (ii) the occurrence of this intrachromosomal amplification throughout the whole CG (India, Nepal, and Bangladesh), and (iii) the estimated emergence of the whole CG around 1850, we may suggest that this amplification was already present in 1850, at the onset of the first reported epidemics of kala-azar. It is noteworthy that *Leishmania* was only discovered half a century later, and for decades, this epidemic disease of the ISC was considered “quinine-resistant malaria” (21). In other words, L. donovani was for decades under pressure from other drugs, such as quinine, and this could also have played a role in the preadaptation.

The untargeted genomic approach here used revealed the importance of genome structure variation for parasite adaptation to drug pressure. Despite our efforts to find new genes or mechanisms implicated in Sb^III^ resistance using whole-genome sequencing (WGS) data, we did not identify any SNP/indel, gene copy number variation, or a consistent somy pattern in the 2 CG strains analyzed (BPK282 and BPK275 [Fig. 3B and C]). On the other hand, two SNPs were found in BPK026 as well as significant modulations of the karyotype occurring rather early during the selection process and partial chromosomal amplifications (Fig. 3D, E, and F). This fits perfectly with the findings of Shaw et al. (22), who showed that during experimental selection of miltefosine resistance, aneuploidy was the first adaptation of the parasite, before the appearance and fixation of SNPs. Similar results were encountered in the context of Sb^III^ resistance in Leishmania major, Leishmania guyanensis, and Leishmania amazonensis (11, 14, 23). Karyotype modulation has been described as a powerful strategy of gene dosage regulation (24), playing a role in environmental stress and in drug resistance (25).

Relative to the two CG strains, aneuploidy changes were more abundant in BPK026 Sb^III^-R, as expected for a less-preadapted or nonpreadapted strain. Among affected chromosomes, two deserved a particular attention, as they were also showing somy changes in one or more CG lines, chromosomes 23 and 31. In BPK026, the increase in the *S* value of chromosome 23 was due to the increase in copy number of a large fragment, containing 140 genes, among others those of the H locus involved in drug resistance and virulence (Data Set S2A). This is in line with extrachromosomal linear amplification, as reported several times in a subgenus of *Leishmania* (26). However, the same fragment of chromosome 23 was shown to be amplified during *in vitro* selection of Sb^III^-resistant Leishmania (Viannia) guyanensis, which is known to be unable to generate intra- or extrachromosomal amplifications (11). Interestingly, the position of the chromosomal breakpoint corresponded to a strand-switch region and to the localization of the centromere of chromosome 23, as described recently for L. major (27). Accordingly, other explanations for the partial amplification of chromosome 23 in our L. donovani strain should be considered, like increase in somy of the whole chromosome, followed by deletion of a small segment. In BPK282 and BPK275, somy changes of chromosome 23 were less pronounced, and preadaptation likely stems from the already present amplification of the H locus. We experimentally confirmed the link between the amplification of the H locus and a higher tolerance to Sb^III^ by overexpression and suggested that among the genes present in the H locus, *MRPA* was the driver (Fig. 4B). The case of chromosome 31 is also interesting, as it contains the gene encoding AQP1, known to be the transporter responsible for the uptake of Sb^III^ (13) and inactive in all parasites of ISC5 because of a 2-nt indel (9). Such an indel was not observed in BPK282 and BPK026 during the Sb^III^ resistance selection, and we did not find any other downregulation mechanism at the genomic level (like the subtelomeric deletion of AQP1 as reported elsewhere [28]) or transcriptomic level (quantitative PCR; qPCR [data not shown]). Further work will be required to understand the meaning of the somy increase of chromosome 31 (25). Bringing together our experimental and phylogenomic data (presence of H locus intrachromosomal amplification in all strains of CG), we believe that MRPA is likely the earlier driver of Sb^III^ resistance. Later on, other genomic adaptations likely appear, such as AQP1 inactivation, which is present only in ISC5 and had not emerged (yet) under our experimental conditions.

Untargeted metabolomics analyses provided results complementary to the genomic study. They clearly supported the preadaptation hypothesis of CG strains and showed that during the Sb^III^ selection process, the metabolism of BPK026 evolved toward levels of CG WT strains. This was best illustrated by GPLs and amino acids. GPLs play an important role in membrane fluidity, as shown earlier (29, 30), and thus in drug resistance since it can act on the absorption of the drug. Amino acids were also modulated, notably proline and arginine (Fig. 5C). Proline levels actually increased in all lines following Sb^III^ resistance selection. This is not unexpected since proline has been shown to enhance cell survival during environmental stresses: in particular, proline is required for volume recovery during osmotic stress responses (31). Increased levels of proline have been observed in other Sb^III^-resistant strains selected *in vitro*, and addition of proline to the culture medium led to increased parasite tolerance to Sb^III^—probably because of its protective role against drug-induced oxidative stress (30). Arginine levels, on the other hand, remained unchanged in the Sb^III^-R CG strains but were increased to the CG level in Sb^III^-R ISC1, perfectly fitting with the preadaptation hypothesis. Arginine is part of the urea cycle, which leads to the synthesis of ornithine, polyamines, and ultimately the antioxidant trypanothione. Increased levels of thiols and of other metabolites linked to thiol production have been associated with better protection against Sb-induced oxidative stress and were demonstrated in other studies on SSG-R parasites (15, 29, 32). Lysine levels also supported the preadaptation hypothesis, remaining unchanged in WT or Sb^III^-R CG strains but decreasing to CG levels in Sb^III^-R ISC1. Further studies are however required to understand the implication of lysine in parasite susceptibility/resistance to Sb^III^.

In summary, we reported for the first time the preadaptation of a whole *Leishmania* population to drug resistance. The preadaptation to Sb^III^ shown here does not mean that all the L. donovani parasites of the CG would cause clinical resistance to SSG, and many of them did indeed show susceptibility to Sb^V^ (as shown in reference 9 and the BPK282 WT in this article). However, under adequate pressure, they could rapidly become resistant, which was not the case with ISC1 parasites. We believe that MRPA amplification (thus increased sequestration of Sb^III^) is a first and important line of defense of the parasites, providing them a grade of tolerance to Sb^III^. Additional adaptive layers—occurring later during the evolution of the CG—were likely required for a higher resistance to Sb^III^ (e.g., AQP1 inactivation, leading to decreased uptake of the drug) or Sb^V^ (e.g., the presence of unique glycans driving the subversion of macrophages) and ultimately for full clinical resistance. Our findings highlight the importance of genetic diversity in the development of resistance and the need for close monitoring of parasite populations before and after implementation of a new drug. Similarly, Research and Development of new drugs should take into account this concept of preadaptation and include a challenge of new compounds to parasites with different preadaptation features.

## MATERIALS AND METHODS

### Strains and culture conditions.

Ten strains representing the diversity of L. donovani in the ISC were used in the present study (9) (Table S1). Promastigotes were maintained in HOMEM (Gibco) supplemented with 20% (vol/vol) heat-inactivated fetal calf serum (FCS) at 26°C. Growth curves of the strains were performed by daily manual counting for 7 days using Uriglass counting chambers (A. Menarini Diagnostics).

### Antimony susceptibility tests.

Sb^III^ susceptibility tests were performed using potassium antimony tartrate (Sigma-Aldrich) as the source of Sb^III^ (36.9%). The EC_50_s were determined on logarithmic-stage promastigotes after 24 h of exposure to Sb^III^ with a resazurin assay as previously described (33). Briefly, promastigotes were exposed to a semilogarithmic concentration range of Sb^III^ from 1 µM to 382 µM. EC_50_s were calculated with GraphPad Prism using a sigmoidal dose-response model with variable slope.

For the Sb^V^ susceptibility assay, primary peritoneal macrophages from Swiss OF1 mice (Charles River, Inc.) were infected with day 7 stationary-phase promastigotes as previously described (33). After 24 h of infection, infected macrophages were washed, and fresh medium containing SSG (Calbiochem) at a concentration range of Sb^V^ between 12.3 and 246.4 µM was added for 5 days before the final wash and Giemsa staining. The number of infected macrophages and the amount of amastigotes per infected macrophage were determined by manual counting. These numbers were used to establish the infection index (% infected macrophages × amastigotes/infected macrophages). The percentage of parasite growth inhibition was calculated as [1 − (InfInd_*x*_/InfInd_0_)] × 100, where InfInd_0_ and InfInd_*x*_ are infection indices of untreated infected cells and cells treated with different SSG concentrations, respectively. EC_50_s were calculated with GraphPad Prism using a sigmoidal dose-response model with variable slope.

### Sb^III^ resistance selection.

For each strain, selection of resistant parasites was initiated in quadruplicates in 200 µl on a 96-well plate. Briefly, in each well, 10^5^ logarithmic-phase promastigotes were incubated for 7 days with Sb^III^ at concentrations ranging from 0.4 to 382 µM following a 2-fold increase. This first step allowed the estimation of the highest concentration tolerated by the parasites at a given time point. The first concentration of the selection process was determined by the highest concentration at which at least 3 replicates were growing. Parasites were then transferred into 5 ml culture medium with the selected concentration of Sb^III^ and maintained for 5 weeks before starting a new selection round. Selection rounds were performed successively with 6, 12, 24, 48, 96, 191, and 382 µM Sb^III^ for BPK026 and 48, 96, 191, and 382 µM Sb^III^ for BPK282 and BPK275. A rapid procedure for Sb^III^ resistance selection (called Sb^III^ flash selection) was also attempted. Wild-type parasites from different genetic groups described in reference 9 were cultured in parallel in triplicates with and without 382 µM Sb^III^ (Table S1). Counting was performed every day for 5 weeks as described above. Parasites were subcultured at 5 × 10^5^ parasites⋅ml^−1^ at day 7 when possible. When the parasite concentration was too low, cells were pelleted and resuspended in fresh medium at a lower Sb^III^ concentration.

### DNA preparation for NGS and data analysis.

Parasites were pelleted after 5 weeks of culture with a given concentration of Sb^III^. DNA isolation was done using QIAamp DNA blood minikit (Qiagen), and the DNA concentration was assessed with the Qubit DNA broad-range DNA quantification kit (Thermo Fisher). Sequencing libraries were prepared with the TruSeq DNA library prep kit (Illumina) according to the manufacturer’s instructions. The libraries were quantified by qPCR using the KAPA Library quantification kit optimized for Roche LightCycler 480 (KAPA biosystems). The libraries were 2× 100-bp paired-end sequenced with the Illumina Hiseq 2000 platform by the Beijing Genomics Institute (BGI) according to standard protocols. Ploidy, single nucleotide polymorphisms (SNPs), and local copy number variations (CNVs) were determined as described by Imamura et al. in 2016 (9) using the BPK282v2 PacBio reference genome accessible via ftp://ftp.sanger.ac.uk/pub/project/pathogens/Leishmania/donovani/LdBPKPAC2016beta/. Sequences are available at the European Nucleotide Archive under accession no. PRJEB22849. Gene identity in the manuscript is given with LdBPK v2 as annotated elsewhere (19). However, LdBPK v1 gene identity, as used at GeneDB (http://www.genedb.org/Homepage), is also mentioned to facilitate comparisons with other studies. Biological and statistical significance of chromosome copy number (*S* value) comparison between samples was done according to two criteria as previously described (25). Briefly, the somy of a given chromosome in a population of cells is known to be a continuous variable with a multimodal distribution of *S* values because of aneuploidy mosaicism. Under this model, the *S* value of homologous chromosomes is thus considered to be significantly different if (i) *S* values differ from more than 0.5 with a somy distribution shift from one mode to another and (ii) the *P* value is ≤10^−5^.

### Metabolite extraction, LC-MS protocol, and data analysis.

For each analyzed sample, metabolites were extracted from four replicates of logarithmic-phase promastigotes (3 days of culture) using a 1:3:1 (vol/vol/vol) chloroform-methanol-water solvent as previously described (29). Samples were analyzed with an Orbitrap Exactive (Thermo Fisher Scientific) mass spectrometer coupled to a 2.1-mm ZIC-hydrophilic interaction liquid chromatography (HILIC) column (Sequent) at Glasgow Polyomics (University of Glasgow, Glasgow, Scotland) as previously described (30). All samples were analyzed in randomized order and in the same analytical batch. Several quality control samples, including solvent blanks, pooled samples, and authentic standard mixtures, were also measured in the same run to verify liquid chromatography-mass spectrometry (LC-MS) stability and allow the identification of contaminants (34). Data analysis was performed in R using the packages XCMS (35) and mzMatch.R (36). The workflow was described in detail previously (29). R was also used to generate heat maps and PCA plots based on all metabolites in the data set with measurements across all samples. For each metabolite, we computed log_2_ fold changes (FC): i.e., the log_2_ ratio of average metabolite abundance between two conditions for comparisons at the level of individual metabolites. The associated *P* value was obtained by performing a Student’s *t* test for each comparison, which was subsequently corrected with the Benjamini-Hochberg algorithm to limit the false-discovery rate (FDR) to 5%. Differences in the amounts of metabolites were considered significant if the log_2_ FC was >1 or <−1 and the corrected *P* value was <0.05.

### Single-gene overexpressers.

Each gene of the H and M loci was overexpressed in BPK026/0 cl5 after cloning in pLEXSY-hyg2 (Jena Bioscience) (Table S2). All genes except the MRPA gene were PCR amplified from BPK282 DNA using Phusion high-fidelity polymerase (NEB) with overhang primers (Table S2) for subcloning in pGEMT (Promega) before final cloning in pLEXSY-hyg2. MRPA was amplified using iProof high-fidelity DNA polymerase (Bio-Rad) with GC buffer plus 10% dimethyl sulfoxide (DMSO) and directly cloned in the pLEXSY-hyg2 using the In-Fusion HD cloning kit (Clontech). Every PCR-amplified sequence was sequenced at the VIB Genetic Service Facility (VIB Genetic Service Facility, Antwerp, Belgium). Promastigotes of BPK026/0 cl5 were then transfected using GenePulser Xcell (Bio-Rad) as described previously (37). Transfected parasites were selected with 50 µg·ml^−1^ of hygromycin (Jena Bioscience) and then maintained with 100 µg·ml^−1^.

10.1128/mSphere.00548-17.5 TABLE S2  Probes and primers. Download TABLE S2, PDF file, 0.1 MB. Copyright © 2018 Dumetz et al. 2018 Dumetz et al. This content is distributed under the terms of the Creative Commons Attribution 4.0 International license.

### Cosmid isolation and transfection.

A cosmid bank of L. donovani MHOM/NP/03/BPK190 (ISC4) had already been prepared in the laboratory of Joachim Clos in Hamburg, Germany (38, 39). Cosmids carrying copies of the H locus (pcosTL-H-Locus) and the M locus (pcosTL-M-Locus) were isolated by colony lifting. Briefly, the cosmid bank was plated on LB agar-ampicillin plates, and a replica of each plate was taken on a nylon membrane (Amersham Biosciences, Inc.). Probes specific for LdBPK_360076800 (MPK1) and LdBPK_230007800 (MRPA) (Table S2) were digoxigenin (DIG) labeled by PCR using DIG-dUTP (Roche), and hybridization was performed overnight at 65°C. The membrane was developed using anti-DIG antibody (Roche) associated with alkaline phosphatase and nitroblue tetrazolium (NBT) as a revealing agent. Colonies with positive signal were isolated from the plate, and colony PCR was used to confirm the presence of the sequence of interest. The two BPK026/0 lines carrying cosmids were obtained by nucleofection of 10 µg of pcosTL H-Locus or pcosTL M-Locus DNA using Basic Parasite Nucleofector kit 1 (Lonza) and Nucleofector II (Amaxa Bioscience) according to the manufacturer’s instructions. Parasites were selected at 50 µg·ml^−1^ and maintained in 100 µg·ml^−1^ of G418 (Sigma).

### RNA extraction and real-time qPCR analysis.

A total of 10^8^ logarithmic-phase promastigotes were pelleted for RNA extraction using the RNAqueous-Micro total RNA isolation kit (Ambion). RNA quantification prior to cDNA synthesis was done using Qubit and the Qubit RNA BR assay (Life Technologies, Inc.). Synthesis of cDNA was performed using Transcriptor reverse transcriptase (Roche) according to the manufacturer’s instructions. qPCRs were run on a LightCycler 480 (Roche) with a SensiMix SYBR No-ROX kit (Bioline) as the SYBR green source; the primers used are displayed in Table S2. Expression levels were assessed with qBase+ (Biogazelle) using for normalization two genes previously shown to be very stable: *SAT* (LdBPK_340035000) (40) and LdBPK_240021200 (25). The expression of both normalization targets was confirmed by qBase+ as stable during the course of the experiment. Data were then rescaled to the BPK026 WT. An increase of 1.5-fold compared to the BPK026 WT was considered significant.
