# Supplementary material for: Molecular Preadaptation to Antimony Resistance in Leishmania donovani on the Indian Subcontinent
Source: mSphere. 2018 Apr 18;3(2):e00548-17. doi: 10.1128/mSphere.00548-17 (PMC5907651; doi:10.1128/mSphere.00548-17)
Supplement: TABLE S1 [file sph002182513st1.pdf]

ISC group	Strain	Treatment outcome	SSG activity index	Used for	H-locus	M-Locus
					Full depth	Full depth
ISC1	MHOM/NP/02/BPK026/0 cl5	SSG definite cure	1	Susceptibility, selection and flash selection	1.59	0.72
	MHOM/NP/02/BPK156/0	SSG definite cure	-	Susceptibility, flash selection	0.97	0.78
	MHOM/NP/02/BPK031/0 cl12	SSG definite cure	1	Susceptibility, flash selection	1.61	0.80
ISC3	MHOM/NP/02/BPK067/0 cl2	-	1	Susceptibility, flash selection	4.30	9.27
ISC4	MHOM/NP/03/BPK206/0 cl10	SSG definite cure	1	Susceptibility, flash selection	2.84	9.10
ISC5	MHOM/NP/03/BPK275/0 cl18	SSG non response	>6	Susceptibility, selection and flash selection	3.85	14.36
	MHOM/NP/02/BPK173/0 cl3	SSG non response	>6	Susceptibility, flash selection	4.50	15.23
ISC6	MHOM/NP/03/BPK282/0 cl4	SSG definite cure	1	Susceptibility, selection and flash selection	4.24	12.78
	MHOM/NP/02/BPK178/0 cl3	SSG definite cure	2	Susceptibility, flash selection	3.96	12.21
ISC9	MHOM/NP/03/BPK294/0 cl1	SSG definite cure	1	Susceptibility, flash selection	4.41	10.53
				Average ISC1	1.39	0.77
				std	0.30	0.03
				Average CG	4.01	11.93
				std	0.52	2.22
