# Supplementary material for: Molecular Preadaptation to Antimony Resistance in Leishmania donovani on the Indian Subcontinent
Source: mSphere. 2018 Apr 18;3(2):e00548-17. doi: 10.1128/mSphere.00548-17 (PMC5907651; doi:10.1128/mSphere.00548-17)
Supplement: TABLE S2 [file sph002182513st2.pdf]

Probes for colony lift of pcosTL-H- and M- locus			
Gene code	Gene name	Primer name	Sequence
LdBPK_230007800	MRPA	MRPA_SBprobe_Fwd	GTCAACAGGCTCGCTTAGGTGTAG
		MRPA_SBprobe_Rev	GGAGACGTTCTGCATGTACAGCG
LdBPK_360076800	MPK1	MPK1_SBprobe_Fwd	GTCTCGAGCTCGGGCGAGTG
		MPK1_SBprobe_Rev	GATGCGCAGCGCACTTATCGG

Primers used for gene expression level analysis			
Gene code	Gene name	Primer name	Sequence
LdBPK_230007600	Hypothetical protein 23 (Hyp23)	Hyp23-For	ATGACACGCTGGAAGGC
		Hyp23-Rev	CAGACGATGAAGGTCAGC
LdBPK_230007700	HTBF	HTBF-For	GAGCTGATAAGGAGCTTCACG
		HTBF-Rev	CAAAGATGAAGACCGCAGAG
LdBPK_230007900	ASS	ASS-For	CGACCGCAACCTGTGGC
		ASS-Rev	GCGACGGGGATGCCC
LdBPK_230007800	MRPA	MRPA-For	TGTGTTTCCGACGATTGC
		MRPA-Rev	GTGACCCGCTTTGTGGAC
LdBPK_360076700	Hypothetical protein 36 (Hyp36)	Hyp36-For	AGCACATGCCACCCAATC
		Hyp36-Rev	ATCCGACGATAGTACCCACAAC
LdBPK_360076800	MPK1	MPK1-For2	GTGGTCGCGCTGCAGAAG
		MPK1-Rev2	CGGCACAACCCCTTCATTG
LdBPK_340035000	SAT	SAT-For	CCCGTATGCTGACAGAGTTGG
		SAT-Rev	GCCGTGGTGAATGAAGAAGTG
LdBPK_240021200	Hypothetical protein chr24	Ld24-For3	GTACCTGATGGTGACGTGG
		Ld24-Rev3	GTCTTTCCCCGGGTGTCTTT

Single genes over-expressors constructs in pLEXSY-hyg2					
Gene code	Gene name	Cloning method	Final vector	Primer name	Sequence
LdBPK_230007600	Hypothetical protein 23 (Hyp23)	sub cloning in pGEMT	pLEXSY-hyg2	BglII-Hyp23-Fwd	accAGATCTATGGCCGGCCAGATCCG
				NotI-Hyp23-Rev	cgcGCGGCCGCTCACCCGCGTTCTCGC
LdBPK_230007700	HTBF	sub cloning in pGEMT	pLEXSY-hyg2	NcoI-HTBF-Fwd	accCCATGGATGCTCAACGAGGTGCA
				NotI-HTBF-Rev	cgcGCGGCCGCCTAAATACCAACCAGA
LdBPK_230007900	ASS	sub cloning in pGEMT	pLEXSY-hyg2	BglII-ASS-Fwd	accAGATCTATGCCTGCAACGGCGAC
				NotI-ASS-Rev	cgcGCGGCCGCTCACAAGCTGCTCGG
LdBPK_230007800	MRPA	In-Fusion	pLEXSY-hyg2	LEXSY-MRPA_Fwd	ACCAGATCTGCCATGGCGACACCAGACTTTGTACG
				LEXSY-MRPA_Rev	GTACCCTTAAGGCTAGTCAGCCGAGGAGATCGACGAAG
LdBPK_360076700	Hypothetical protein 36 (Hyp36)	sub cloning in pGEMT	pLEXSY-hyg2	NcoI-Hyp36-For	accCCATGGATGAGTTCTGCGCTGCAAC
				NotI-Hyp36-Rev	cgcGCGGCCGCTCACTCCTCTTCGAGAACAC
LdBPK_360076800	MPK1	sub cloning in pGEMT	pLEXSY-hyg2	NcoI-MPK1-For	accCCATGGATGACCTCCTATGGCATCGAC
				NotI-MPK1-Rev	cgcGCGGCCGCCTAATACACGTCTGTTATG

Red sequence: Restriction site needed to clone inside pLEXSY-hyg2

Blue sequence: Homology sequence to pLEXSY for in-fusion cloning

Minor letters are protective necleotides
